# Effect of early glycemic control on HbA1c tracking and development of vascular complications after 5 years of childhood onset type 1 diabetes: Systematic review and meta‐analysis

**DOI:** 10.1111/pedi.12850

**Published:** 2019-04-24

**Authors:** Veena Mazarello Paes, Jessica K. Barrett, David C. Taylor‐Robinson, Heather Chesters, Dimitrios Charalampopoulos, David B. Dunger, Russell M. Viner, Terence J. Stephenson

**Affiliations:** ^1^ Great Ormond Street Institute of Child Health University College London London UK; ^2^ Department of Paediatrics University of Cambridge Cambridge UK; ^3^ MRC Biostatistics Unit University of Cambridge Cambridge UK; ^4^ Institute of Population Health Sciences, University of Liverpool Liverpool UK; ^5^ Wellcome Trust/MRC Institute of Metabolic Sciences, University of Cambridge Cambridge UK; ^6^ The Royal College of Paediatrics and Child Health London UK

**Keywords:** childhood‐onset, complications, glycemic control, risk, T1D

## Abstract

**Objective:**

A systematic review and meta‐analysis was conducted to investigate if glycemic control measured by glycated hemoglobin (HbA1c) levels near diagnosis are predictive of future glycemic outcomes and vascular complications in childhood onset type 1 diabetes (T1D).

**Methods:**

Evidence was gathered using electronic databases (MEDLINE, EMBASE, Web of Science, CINAHL, Scopus, and Cochrane Library up to February 2017) and snowballing techniques. Studies investigating the association between the exposure “early glycemic control” and main outcome: “tracking of early control” and secondary outcome: risk of future complications; in children and young people aged 0 to 19 years at baseline; were systematically double‐reviewed, quality assessed, and outcome data extracted for synthesis and meta‐analysis.

**Findings:**

Five studies (N = 4227 participants) were eligible. HbA1c levels were sub‐optimal throughout the study period but tended to stabilize in a “track” by 6 months after T1D diagnosis. The group with low HbA1c <53 mmol/mol (<7%) at baseline had lower long‐term HbA1c levels than the higher HbA1c group. The estimated standardized mean difference between the sub groups showed a reduction of HbA1c levels on average by 1.6% (range −0.95% to −2.28%) from baseline. Only one study investigated the association between early glycemic control and development of vascular complications in childhood onset T1D.

**Interpretations:**

Glycemic control after the first few months of childhood onset T1D, remains stable but sub‐optimal for a decade. The low and high HbA1c levels at baseline seem to “track” in their respective tracks during the 10‐year follow‐up, however, the initial difference between groups narrows over time.

**PROSPERO:** CRD42015024546 http://www.crd.york.ac.uk/PROSPERO/display_record.asp?ID=CRD42015024546

**ABBREVIATIONS**
DCCT: The Diabetes Control and Complications Trial
EPPI: Evidence for Policy and Practice Information
FE: Fixed effects model
HbA1c: Hemoglobin A1c
RE: Random effects model
PROSPERO: International Prospective Register for systematic Reviews
T1D: Type 1 diabetes

## 1 INTRODUCTION

Glycated hemoglobin (HbA1c) levels, a measure for glycemic control is the main predictor of long‐term type 1 diabetes (T1D) outcomes.^1^, ^2^, ^3^ HbA1c levels are highest at diagnosis, but improve after insulin treatment and remain stable in most T1D patients. However, a few find it challenging to maintain good glycemic control despite targeted or intensive interventions, as they go through various stages in life.^4^, ^5^

Studies mainly in adults have shown a link between poor glycemic control in the early phase following T1D diagnosis and long‐term HbA1c levels, with an increased risk of developing vascular complications and mortality.^6^, ^7^ The risk of vascular complications is likely to be greater for childhood onset T1D, because of a longer duration of glycemic exposure^8^ and pathophysiological factors, such as reduced insulin sensitivity and psychosocial behaviors, such as insulin omission.^9^, ^10^, ^11^ For childhood onset T1D, some observational studies indicate an association between poor glycemic control within 1 or 2 years of diagnosis and vascular complications in later life.^12^, ^13^, ^14^ Others suggest that mean HbA1c levels nearer to diagnosis are predictive of HbA1c levels in the subsequent years, even lifetime, regardless of the type of insulin regimen.^15^, ^16^, ^17^ This phenomenon, also known as glycemic “tracking,” is poorly understood.^18^ It is unclear exactly when and in whom the phenomenon of “tracking” of HbA1c occurs in childhood onset T1D and if it is because of the natural history of T1D. It is therefore important to investigate the evidence on this phenomenon to identify if there exists a window period in the initial phase of T1D diagnosis, during which appropriate resources could be mobilized to deliver targeted interventions to those at risk of developing poorer long‐term glycemic outcomes and vascular complications.

The purpose of our study was to carry out a systematic review and meta‐analysis of the evidence assessing the impact of early glycemic control in children (followed for at least 5 years from diagnosis) on tracking of early control and the risk of developing vascular complications.

## 2 METHODS

This review is part of a series of systematic reviews of evidence on the effects of early glycemic control in childhood onset T1D. The review protocol was registered in PROSPERO (Registration number: CRD42015024546) and a detailed protocol published.^19^ We followed the review methods for the rigorous conduct and reporting of systematic reviews for policy and practice as described by the Evidence for Policy and Practice Information (EPPI) Centre^20^ which are as per PRISMA guidelines.^21^

### 2.1 Search strategy

A refined search strategy was designed after a number of initial iterative scoping searches, with input from experts in the field to maximize capturing of key publications. Three sets of search terms were used relating to population (children and young people diagnosed with T1D), exposure (terms to capture observational, intervention, qualitative studies, and review articles relating to early diabetes control) and outcome (complications, mortality, glycemic tracking i.e., metabolic memory) (Additional File 1).

Six electronic databases: (MEDLINE and EMBASE through OVID, Web of Science through Thompson Reuters, CINAHL Plus through EBSCO, Scopus through Elsevier, and the Cochrane Library), were double searched in parallel by HC & VMP from inception to December 2014 and updated in February 2017 by using a combination of free text and Thesaurus or MeSH terms (Additional File 2). No time‐period or language restrictions were applied. All identified articles from electronic databases were imported into Endnote and de‐duplicated for further review. This was supplemented by hand‐searching of reference lists of studies and reviews, gray literature, personal databases and contacting experts and authors of included studies for additional or unpublished data.

### 2.2 Study selection

Interventional and observational studies with a follow‐up of ≥5 years from diagnosis of T1D which described and quantified the association between early glycemic control (defined as glycemic control within 2 years of diagnosis of T1D) AND long‐term glycemic tracking (defined as settling of HbA1c levels into long‐term tracks of either > or <7% ie, 53 mmol/mol) and risk of future complications in children and young people aged 0 to 19 years at baseline were included (Additional File 3).

In addition to running electronic database searches in parallel (HC and VMP), sub‐samples of papers were double‐reviewed (DC and VMP), at each stage of the review process (title and abstract screening, data extraction and quality assessment). The interrater reliability for study selection was substantial.^22^ Full texts of abstracts appearing to meet the inclusion criteria were retrieved and their status was recorded in a pre‐piloted excel spread‐sheet, which included specific study details and reasons for exclusion (for excluded studies). No foreign language papers were identified. Articles were re‐examined (DC and VMP) if there was uncertainty about inclusion criteria and disagreements were resolved at team meetings.

### 2.3 Data extraction

Data from included studies were extracted, analyzed, and synthesized by one reviewer (VMP). A proportion of shortlisted studies were also independently double reviewed and data extracted (DC and RA). From observational studies, data on HbA1c levels were extracted at all available time points from diagnosis. Data on HbA1c tracking and the association between early glycemic control and chronic complications or markers of chronic complications at follow‐up were extracted (Additional File 4). Authors of included studies were contacted for clarity and additional information on HbA1c tracking data where necessary. The main outcome of interest was tracking of early glycemic control based on HbA1c measurements as percentage (DCCT) and/or mmol/mol (International Federation of Clinical Chemistry) units. The secondary outcome of interest was the impact of early glycemic control on the development of micro and macro vascular complications during the long‐term follow‐up period.

### 2.4 Quality assessment

The quality of included studies was assessed independently by two reviewers (DC and VMP) using the quality assessment criteria by the EPPI Centre.^20^ Any disagreements were resolved by consensus. Scores were based on six items focusing on both internal and external validity (Additional File 5). Observational studies were classified as high (≥5), intermediate^3^, ^4^ or low (≤2) quality based on the number of quality criteria met out of a maximum assessment score of six.

### 2.5 Statistical analysis

Information extracted from included studies were summarized through descriptive narrative synthesis and meta‐analysis.^23^ All statistical analyses were conducted by one reviewer (VMP) and were verified by a second reviewer (JB). The sample size, mean HbA1c measurements and SD or SE were available at population level and/or for categorized low and high HbA1c groups. Where not reported, the SE of the study at each time point was calculated using the reported SD and the group sample sizes. Baseline period included 3 to 6 months from T1D diagnosis. Mean HbA1c levels at diagnosis was not included in the main meta‐analysis as by definition they were measured prior to exposure of glycemic control with insulin therapy. The effect sizes and their SE were divided with SD to obtain standardized mean differences (SMD).^24^

The primary outcome was the population mean HbA1c level at baseline (0, 3, and 6 months of diagnosis), 1, 2, 3, 5, 7, and 10 years follow‐up. A further primary outcome was the difference in HbA1c levels between the low HbA1c (<7% at baseline) group (considered the “treated/exposed” group) and the high HbA1c group (≥7% at baseline) (the “control” group), reported as standardized mean differences. If multiple measurements of HbA1c were reported at follow‐up then these measures were combined within each study before meta‐analysis. Heterogeneity between studies was expected and therefore both fixed effects (FE, inverse variance) and random effects (RE, Dersimonian, and Laird) models were used to pool the effect sizes and reported using forest plots.^25^ The heterogeneity between studies was assessed using the *χ* ^2^ test for heterogeneity and *I* ^2^ statistics.^26^ The meta‐analyses were carried using the metan command in STATA 15, StataCorp, College Station, Texas.

For glycated hemoglobin, the estimated pooled standardized mean differences were converted into absolute units, to facilitate clinical interpretation, by multiplying the estimate by the pooled SD of all included studies of the meta‐analysis.

Furthermore, the long‐term population average HbA1c trajectory from each study was plotted alongside the overall estimate at all‐time points of follow‐up obtained from the meta‐analysis. The trajectories of HbA1c sub groups (low v/s high) in each study were also plotted.

The robustness of the meta‐analysis to the choice of meta‐analysis model was assessed by comparing FE and RE pooled standardized effect sizes. In a sensitivity analysis we excluded studies in pre‐school children.

Assessing publication bias using the funnel plots, the Begg's rank correlation test or the Egger's linear regression test was deemed inappropriate as there were insufficient studies included in the review.

Because of the small number of included studies, meta‐regression was not appropriate to explore heterogeneity between studies or to investigate if there were other potential factors that could be independently associated with long‐term glycemic control. A minimum of 10 studies per study level parameter would be needed for meta‐regression.

Only one included study assessed the association of micro and macro‐vascular complications with early glycemic control, which precluded a meta‐analysis and results of which were narrated separately.

## 3 RESULTS

**Figure 1:**
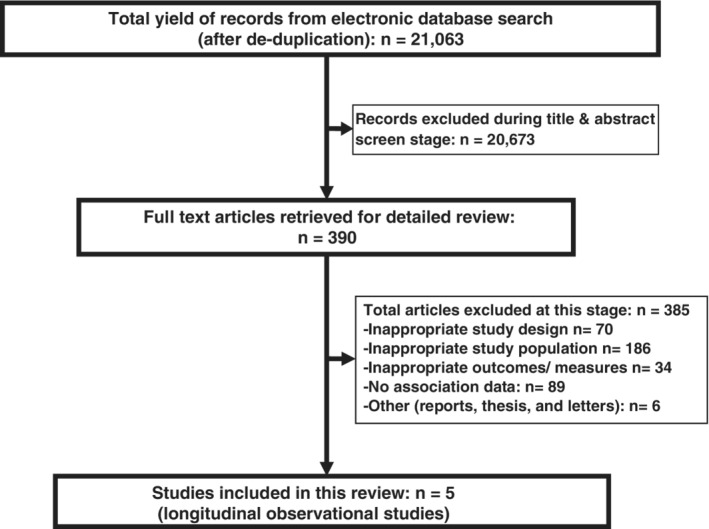
Stages of systematic review of evidence on long‐term glycaemic control

The literature search strategy on glycemic control in childhood onset T1D identified articles from individual databases (Medline through OVID, n = 14 688; Embase through OVID, n = 843; Web of Science through Thompson Reuters, n = 2734; CINAHL Plus through EBSCO, n = 1185; Scopusthrough Elsevier, n = 2837 and Cochrane library, n = 4052). After de‐duplication 21 063 articles were screened, out of which 390 were shortlisted for full review (Figure 1). There was good agreement between reviewers on identifying abstracts for full text review. A total of 385 studies were excluded from the systematic review and meta‐analysis for reasons shown in Figure 1. Five fairly recent studies^24^, ^27^, ^28^, ^29^, ^30^ conducted in developed countries (Israel, Scotland, Sweden, and USA) with a total of 4227 participants met the inclusion criteria of the systematic review. The studies investigated national,^24^ regional,^27^ Children's hospital,^29^ academic medical centre^30^ and clinic^28^ level data.

### 3.1 Characteristics of included studies

The Swedish cohort study^24^ consisted of 1543 children and adolescents (920 males) from two nationwide population‐based Swedish registries (Swedish Pediatric Quality Registry and Swedish National Diabetes Register) covering a period from year 2000 to 2010. The mean age at diagnosis was 13.9 (range 5.0‐19.0) years and the mean follow‐up was for 7.1 ± 2.5 (range 1.0‐12.0) years. The study investigated whether high mean HbA1c values 3 to 15 months after diagnosis of T1D in childhood was associated with future glycemic control, albuminuria and retinopathy in early adulthood.

The American study^29^ prospectively investigated, between the years 1993 and 2009, whether age at diagnosis, gender, ethnicity, diagnostic era (year of diagnosis) and type of insulin therapy were associated with tracking of glycemic control at 5 years follow‐up post diagnosis of T1D. A total of 2218 (1166 males) mainly non‐Hispanic Caucasian (86.1%) children and adolescents participants with a mean age of 9.0 ± 4.1 years at diagnosis (range 0‐20 years), were identified from the Children's Mercy Hospital T1Ds in pediatrics database, USA. Insulin therapy (split regimen dosing, multiple daily injections and continuous subcutaneous insulin infusion) and diagnostic methods used to analyze HbA1c varied during the study period. Information on the socio‐economic status and T1D history in family was not reported.

The other American study^30^ followed 138 children (71 males and 91.5% white) at an academic medical center of Pediatric Endocrinology/Diabetology at Riley Hospital for Children, Indiana, USA and investigated whether long‐term HbA1c differed as a result of receiving diabetes related education during the years 1998 to 2002. The mean age at diagnosis was 6.8 ± 3.3 years (age range: 1.1‐13.9 years). Details of insulin therapy was not reported.

The Scottish study^27^ retrospectively investigated HbA1c tracking among 155 children (74 males), aged ≤16 years (range 0 to 16 years), from the regional database of the National Health Service (NHS) Highland Pediatric diabetic services followed for a median of 4.10 (range 0 to 15.0) years from diagnosis between the years 1993 and 2012. The cohort had limited ethnic diversity, low use of intensive insulin therapy and no use of pump therapy.

The Israeli study^28^ was a retrospective observational study, investigating HbA1c tracking in 173 mainly Jewish (84.4%) preschool aged children (84 males) aged 0.5 to 6.5 years at diagnosis between 1993 and 2009 at a tertiary level diabetes clinic in Israel, with a median T1D duration of 4.3 years (range 1 to 11 years) and followed up for 7 years from T1D onset. All patients were advised on carbohydrate counting, required to perform >6 self‐ blood glucose measurements per day and both multiple daily injections and insulin pumps were used.

Further details of the data extracted from the five studies included in the systematic review are in Table 1.

**Table 1:** Description of longitudinal studies investigating the impact of early glycaemic control on long‐term HbA1c and risk of complications in childhood onset T1D

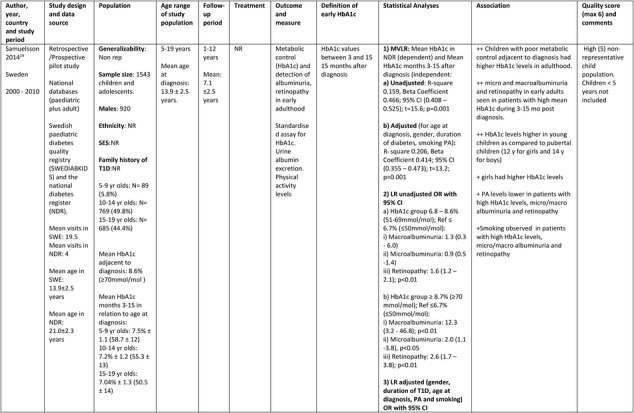
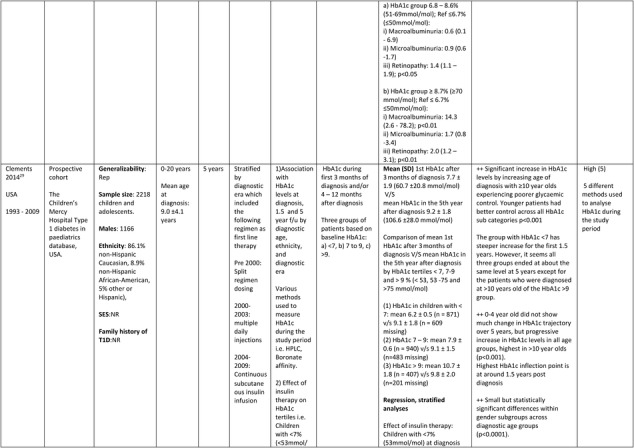
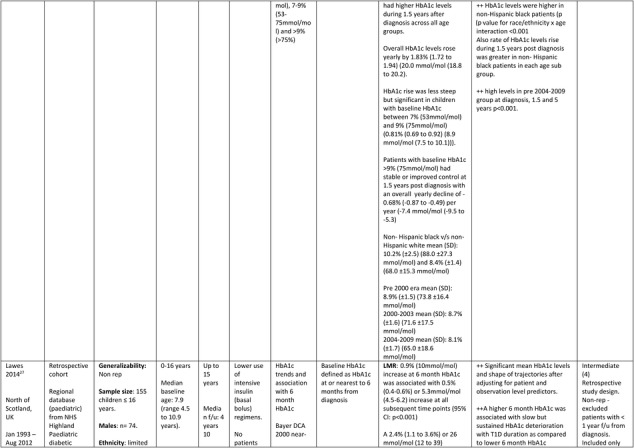
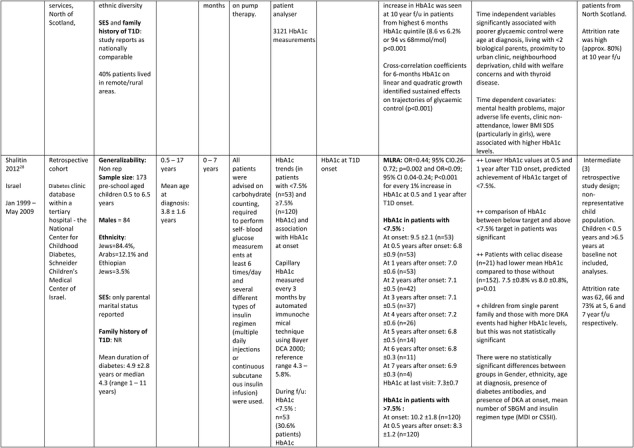
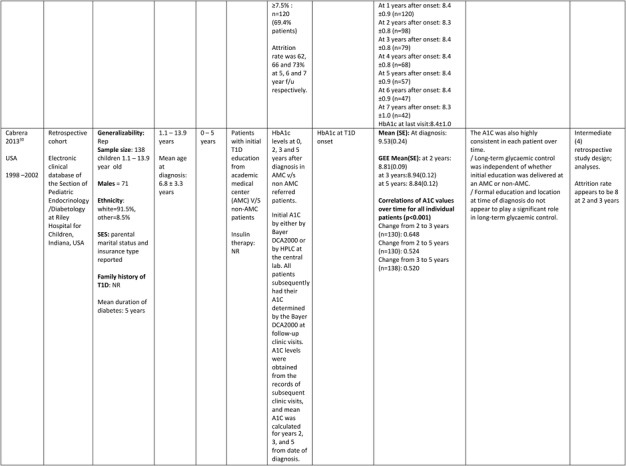

Abbreviations: BMI SDS, body mass index SD score; CI, confidence intervals; GEE, generalized estimating equation; LMR, Linear multilevel regression; LR, logistic regression; MVLR, Multivariate linear regression; NON REP, non representative of general population; MLRA, multiple logistic regression analysis; PA, physical activity; OR, odds ratio; SD/E, standard deviation/error; T1D, type 1 diabetes; ++, statistically significant positive association; + or ‐, statistically non‐ significant positive or negative association.

### 3.2 Study quality

The quality of the observational studies was intermediate to high. Two studies were assessed to be “high” quality with a score of five each^24^, ^29^ and the other three were of “intermediate” quality, with scores of four^27^, ^30^ and three^28^ out of a possible score of six respectively. No studies included in the review were of low quality.

### 3.3 Early HbA1c levels and long‐term tracking of glycemic control

All five studies included in the review assessed the association between early glycemic control and later HbA1c levels. Population mean HbA1c was available at various follow‐up time points (0, 3, 6, 12, 24, 36, 48, 60, 72, 84, 96, 108, 120, 132, and 156 months after T1D diagnosis). In addition, four studies provided data on the association between early glycemic control and later HbA1c levels within sub groups of low and high HbA1c identified at baseline.^24^, ^27^, ^28^, ^29^

To study the impact of early glycemic control on later HbA1c levels, data from all five studies could be pooled in the review. The number of studies reporting the effect during each time point of the study period varied. All studies reported sub‐optimal estimated mean long‐term glycemic control at all of the investigated time points during the 10‐year follow‐up period. The sample size varied from 25 to 2218 and the study periods ranged between years 1993 and 2012. After using the population mean HbA1c and SE in the FE & RE models, the estimated pooled magnitude of the mean HbA1c levels (95% CI) was suboptimal at 11.56% (CI: 11.46, 11.66%) at diagnosis, 7.74% (CI: 7.68, 7.80%) after 3 months 7.61% (CI: 7.47, 7.76%) after 6 months, 7.79% (CI: 7.71, 7.87%) after 1 year, 7.90%(CI: 7.83, 7.98%) after 2 years, 7.94% (CI: 7.86, 8.03%) after 3 years, 8.57% (CI: 8.49, 8.65%) after 5 years, 7.99% (CI: 7.85, 8.12%) after 7 years and 8.59% (CI: 8.24, 8.94%) after 10 years of T1D diagnosis.

**Figure 2:**
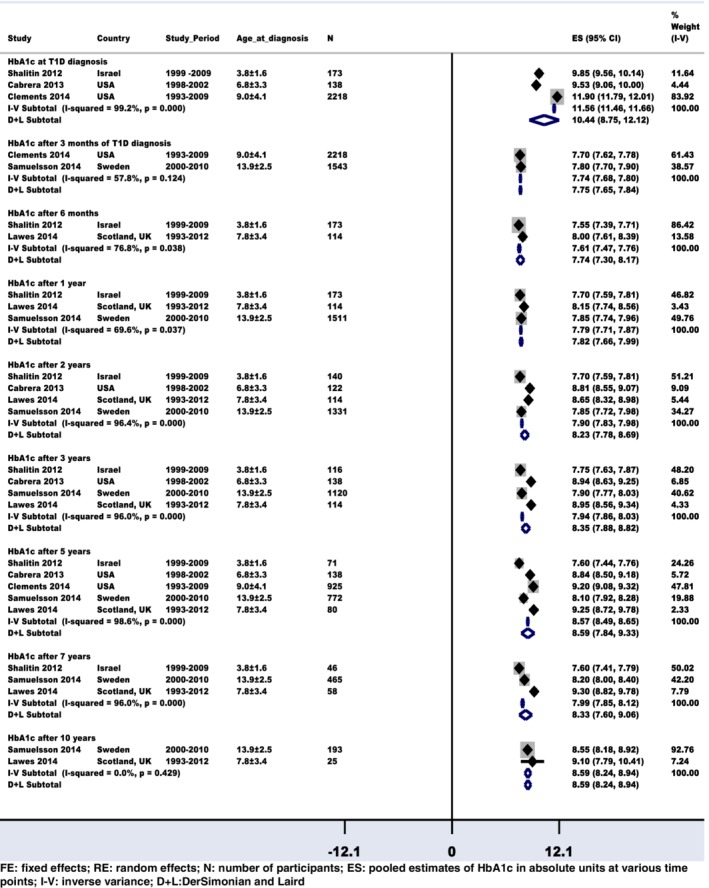
Summary of fixed effects and random effects models: Pooled estimates of overall glycaemic control at follow‐up

The pooled results comparing the effect size results of the FE and RE models were presented in forest plot (Figure 2) and the overall effect estimates were also presented in a graph (Supplementary Figure 2). There was variation in glycemic control between countries in children and adolescents during the 10‐year study period. The test for heterogeneity between studies was significantly high (*I* ^2^ > 69%) at almost all of the follow‐up time points in the meta‐analysis (χ^2^ *P* < 0.05).

Further exploratory sub‐group analysis indicates that heterogeneity was consistently high between studies, countries and populations.

**Figure 3:**
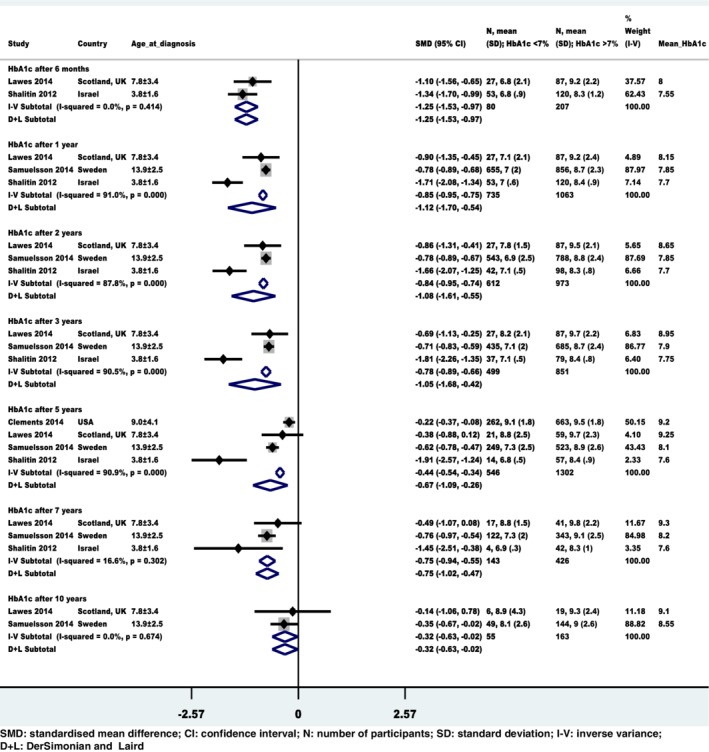
Summary of fixed effects and random effects models: Estimated standardized mean difference of glycated hemoglobin (HbA1c) levels with 95% confidence interval between the low (exposed to glycaemic control) and high (unexposed to glycaemic control) HbA1c groups during various time‐points of follow‐up

For the assessment of early glycemic control (low and high HbA1c identified at baseline) and what followed at various time points during the study period, there were four studies with data that could be pooled in the review. The HbA1c levels of the low HbA1c group showed better improvement than the high HbA1c group during the study period. The low and high HbA1c levels at baseline seem to “track” in their respective tracks during the 10‐year follow‐up however, the initial difference between groups narrows over time (Figure 3).

From the FE meta‐analysis, the pooled standardized difference in mean HbA1c levels between patients in the low HbA1c group and those in the high HbA1c group with 95% CI was significant at −1.25 (−1.53, −0.97) after 6 months, −0.85 (−0.95, −0.75) after 1 year, −0.84 (−0.95, −0.74) after 2 years, −0.78 (−0.89, −0.66) after 3 years, −0.44 (−0.54, −0.34) after 5 years, −0.75 (−0.94, −0.55) after 7 years and − 0.32 (−0.63, −0.02) after 10 years of T1D diagnosis. The treatment effect in absolute units was a reduction of HbA1c levels on average by 1.6% (range − 0.95 to −2.28%) from baseline, which may be clinically relevant (Table 2).

**Table 2:** Summary of pooled standardized mean differences in HbA1c levels between low and high HbA1c groups

T1D duration	MA with all four studies			Sensitivity MA (after excluding study in pre‐school children)		
	SMD (95% CI)	HbA1c % (95% CI)	Heterogeneity (*I* ^2^)	SMD (95% CI)	HbA1c % (95% CI)	Heterogeneity (*I* ^2^)
After 6 months of T1D diagnosis	−1.25 (−1.53, −0.97)	−2.28% (−2.79%, −1.77%)	0.0%, *P* = 0.41	−1.10 (−1.56, −0.65)	−2.37% (−3.35%, −1.40%)	0.0%, *P* = 0.01
After 1 year of T1D diagnosis	−0.85 (−0.95, −0.75)	−2.02% (−3.06%, −0.97%)	91.0%, *P* = 0.001	−0.79 (−0.89, −0.69)	−1.74% (−1.96%, −1.52%)	0.0%, *P* = 0.61
After 2 years of T1D diagnosis	−0.84 (−0.95, −0.74)	−1.76% (−2.63%, −0.90%)	87.8%, *P* = 0.001	−0.78 (−0.89, −0.67)	−1.48% (−1.69%, −1.27%)	0.0%, *P* = 0.73
After 3 years of T1D diagnosis	−0.78 (−0.89, −0.66)	−1.75% (−2.80%, −0.70%)	90.5%, *P* = 0.001	−0.71 (−0.83, −0.59)	−1.48% (−1.73%, −1.23%)	0.0%, *P* = 0.93
After 5 years of T1D diagnosis	−0.44 (−0.54, −0.34)	−1.25% (−2.03%, −0.48%)	90.9%, *P* = 0.001	−0.41 (−0.73, −0.09)	−0.90% (−1.60%, −0.20%)	85.7%, *P* = 0.001
After 7 years of T1D diagnosis	−0.75 (−0.94, −0.55)	−1.19% (−1.62%, −0.74%)	16.6%, *P* = 0.30	−0.72 (−0.92, −0.53)	−1.48% (−1.89%, −1.09%)	0.0%, *P* = 0.40
After 10 years of T1D diagnosis	−0.32 (−0.63, −0.02)	−0.95% (−1.87%, −0.06%)	0.0%, *P* = 0.67	−0.32 (−0.63, −0.02)	−0.95% (−1.87%, −0.06%)	0.0%, p = 0.67

Abbreviations: CI, confidence interval; HbA1c, glycated hemoglobin; MA, meta‐analysis; SMD, standardized mean difference; T1D, type 1 diabetes.

The study in pre‐school aged children (mean age at diagnosis 3.8 ± 1.6 years) showed better control than the other studies with older children.^28^ The heterogeneity levels were significantly high (*P* = 0.001) at 1, 2, 3, and 5 years after diagnosis and were lower at follow‐up time points 0.5, 7, and 10 years after diagnosis (*P* > 0.7) in the meta‐analysis.

The meta‐analysis was repeated after excluding the study in pre‐school aged children (Supplementary Figure 1). The pooled standardized mean difference in HbA1c levels between patients in the low HbA1c group and those in the high HbA1c group with 95% CI was slightly lower at −1.10 (−1.56, −0.65) after 6 months, −0.79 (−0.89, −0.69) after 1 year, −0.78 (−0.89, −0.67) after 2 years, −0.71 (−0.83, −0.59) after 3 years, −0.41 (−0.51, −0.30) after 5 years, −0.72 (−0.92, −0.53) after 7 years and − 0.32 (−0.63, −0.02) after 10 years of T1D diagnosis. The treatment effect in absolute units was a reduction of HbA1c levels on an average by 1.49% (range − 0.90 to −2.37%) from baseline. The test for heterogeneity showed improved results and was significantly high only at 5 years after diagnosis (*P* = 0.001) in the meta‐analysis (Table 2).

Comparing the long‐term HbA1c trajectories between studies revealed that the Israeli study in pre‐school children yielded better long‐term control (Supplementary Figure 2). Individual study results suggest that early glycemic control tracks during the follow‐up in the initially low and high HbA1c groups (Supplementary Figure 3).

Because there were only five studies in the review, we could not assess publication bias using the funnel plot, the Begg adjusted rank correlation test or the Egger test as there was insufficient power to distinguish real asymmetry from random chance.

### 3.4 Association of early HbA1c levels and complications risk

Only one longitudinal study^24^ investigated the association of early glycemic control and future complications and met the inclusion criteria for the systematic review. The study, adjusted for gender, T1D duration, age at diagnosis, physical activity, and smoking; and reported that Swedish children with higher mean HbA1c levels of ≥8.7% (≥70 mmol/mol), 3 to 15 months after diagnosis were significantly more likely to develop macroalbuminuria (OR: 14.3, 95% CI: 2.6‐78.2, *P* < 0.01), microalbuminuria (OR: 1.7, 95% CI: 0.8‐3.4, *P* < 0.05) and retinopathy (OR: 2.0, 95% CI: 1.2‐3.1, *P* < 0.01) in early adulthood (mean age: 21 ± 2.3 years, range: 18‐29 years). The study also highlighted the lack of physical activity, smoking, and female gender as predictors of poor glycemic control. However, the role of insulin therapies and other social and family factors on these observations was not reported.

## 4 DISCUSSION

We identified five longitudinal studies investigating the impact of early glycemic control on long‐term glycemic control in children and adolescents (<19 years) followed from diagnosis of T1D. In the meta‐analysis of all included five studies, the overall mean HbA1c levels in all studies were sub‐optimal at all follow‐up time points.

The meta‐analysis of the four studies comparing initially low v/s high HbA1c groups, indicates that the low HbA1c group showed overall slightly improved control than the high HbA1c group during the study period. In addition, the meta‐analyses suggests that the overall glycemic control was stable in a “track” after 6 months of childhood onset T1D diagnosis. The low and high HbA1c levels at baseline also seem to “track” in their respective tracks during the 10‐year follow‐up. However, the initial difference between groups narrows over time. The number of participants in the low HbA1c group was small and this may have influenced the power to detect group differences.

Three of the included studies were of intermediate quality while the remaining two were of high quality in reporting potential biases. We adhered to strict systematic review procedures for study selection, data extraction and reporting to minimize reviewer related biases. The age ranges and sample sizes varied between studies which may have influenced the heterogeneity seen in the pooled estimates of long‐term glycemic control. Heterogeneity was reduced when the study in pre‐school children was excluded from the meta‐analysis.

All studies included in the systematic review were conducted in developed countries, which had dissimilar health system models and this may have impacted the long‐term glycemic outcomes. The study period was between years 1993 and 2012, during which period understanding of the disease and diagnostic methods for HbA1c testing improved. This may have affected the interpretation of the HbA1c measurements. Also, several changes were implemented during this period in diabetes care, practice and management, through introduction of novel fast acting insulin formulations, intensive insulin treatment and educational interventions. These and the improved diagnostic and clinic factors may have played a role in improving the overall glycemic trajectories in the participants as reported by other studies.^31^, ^32^

The sub‐optimal HbA1c control estimated in the meta‐analysis during the follow‐up period may be because of more participants with higher HbA1c levels, age,^33^ endogenous and exogenous factors or biological variation in the glycation phenotypes of children,^34^, ^35^, ^36^ psychological factors particularly in older children.^37^, ^38^ These are all factors which may also have increased the risk of developing or progression of micro and macrovascular complications in those children as a consequence of those higher HbA1c levels.^39^

The DCCT cohort were able to achieve HbA1c levels of 7% (53 mmol/mol)^40^ as compared with 8.3% (66 mmol/mol) achieved among more than 25 000 patients from USA^41^ and 8.7% (70.1 mmol/mol) achieved by the pediatric population of England and Wales in the UK.^42^ This highlights the fact that, outside of a clinical trial, achieving glycemic targets remains difficult. Hence robustly identifying factors early in the life course of childhood onset T1D that influence future glycemic control and risk of complications remains an important clinical research goal.

Only one study provided evidence that albuminuria and retinopathy were associated with high mean HbA1c of ≥ 8.6% (≥70 mmol/mol) between 3 and 15 months after diagnosis of T1D.^24^ This is consistent with findings by other studies, which did not meet our inclusion criteria.^6^, ^17^, ^43^, ^44^ It would be highly relevant for determining future prognosis, if these outcomes could be confirmed in future studies.

Cardiovascular disease is the major cause of death in T1D patients. Pre‐symptomatic cardiovascular disease is evident in 100% of young adults with T1D^45^ and there is evidence of accelerated atherosclerotic processes^46^, ^47^ and increased severity of cardiovascular disease^48^ at an earlier age compared to the general population. Landmark trials show that intensive insulin therapy reduces cardiovascular events in adults.^6^, ^49^ Although differences in HbA1c account for most of this benefit, multivariate analyses suggest that part of the reduced risk is mediated by reduction in the incidence of diabetic renal disease.^50^ In children and young people with T1D, atherosclerosis is present to a greater extent^51^ and the prevalence of cardiovascular risk factors is greater^52^, ^53^ than in the general population. Diabetic nephropathy incidence accelerates during adolescence.^54^ These are all strong indicators of a greatly elevated risk for future vascular diseases. There is currently no evidence base for the effectiveness of ACE Inhibition or statin treatments in adolescents with T1D although, the important AdDIT Trial may inform practice in the coming years.^55^ Therefore currently, in order to reduce vascular complications risk, the importance of achieving good glycemic control is arguably greater in childhood compared to adult T1D populations.

The meta‐analysis indicates that the overall glycemic control stabilizes in a “track” after 6 months of childhood onset T1D diagnosis and pre‐school aged children had better control throughout the follow‐up period. Furthermore, the low and high HbA1c levels at baseline also seem to have metabolic memory, which shows HbA1c “tracking” during the 10‐year follow‐up despite differences between the high and low groups. This suggests there may be benefits of having good control during the initial few months of diagnosis. However, as these five studies report temporal associations, an experimental study of an intervention soon after diagnosis would be required to prove that better early control results in better later control. This review may also indicate a short window of opportunity to intervene and improve long‐term glycemic outcomes. It may therefore be beneficial to develop clinical and educational strategies to identify and deliver targeted interventions during this early phase to those at risk of having poor glycemic control and to ensure that the HbA1c targets are maintained in the long‐term. There is currently no evidence on effectiveness and timing of focused clinical interventions targeted at changing these tracks.^18^ It would be useful to gather this evidence and to explore further the mechanisms of this phenomenon in order to deliver best care to newly diagnosed children and adolescents. The findings of this review would be useful to policy makers, health professionals and T1D patients to focus on designing interventions to prevent sub‐optimal glycemic outcomes and decrease the risk of developing micro and macro vascular complications.

### 4.1 Strengths and limitations of the review

The many strengths of this study include, being to our knowledge, the first systematic review and meta‐analysis to rigorously investigate published and unpublished literature on the association of early glycemic control in childhood onset T1D with glycemic tracking and future risk of complications. Furthermore, this is the first review to rigorously and systematically search and review all available evidence as per pre‐set inclusion/exclusion and quality assessment criteria. We have taken utmost care to minimize study selection, reviewer related and publication bias. All of the included studies were intermediate to high quality.

But, there are limitations to this systematic review which need to be considered. The diabetes diagnosis, care, and HbA1c outcome measures have evolved over the years and were not uniform across studies. There was considerable heterogeneity between studies. The comparable follow‐up data was not available beyond 10 years. We were unable to investigate if other factors may have confounded the findings. The small number of studies and the short duration of follow‐up in studies may have masked the true association with long‐term glycemic control. Although we made every effort to search for unpublished and gray literature, we may have missed some that remain unreported because of unethical practices in reporting or publication bias. The results of our study may not be generalizable as they were mainly conducted in developed countries with varied health care system models.

### 4.2 Review updating plans

The review will be updated if significant new evidence becomes available and results of the update review will be disseminated through peer‐reviewed publications, conference presentations and at meetings.

## CONFLICT OF INTEREST

No potential conflict of interest was reported by the authors.

## AUTHORS CONTRIBUTION

VMP was the lead reviewer, designed the study, developed the study protocol, created the search strategy, searched electronic databases for literature, extracted the data, co‐ordinated with authors of included studies for additional information, analyzed the evidence, drafted the report and is responsible for the article. JB and DTR participated in the study design, contributed to the statistical analysis design and helped revise the manuscript. HC participated in the study design, contributed to the literature search and helped revise the manuscript. DC participated in the study design, contributed to the double review of a proportion of articles and helped revise the manuscript. DD advised on the project, commented on the analyses and helped revise the manuscript. RV advised on the project, participated in the study design, commented on the analyses and helped revise the manuscript, TS participated in the study design and helped revise the manuscript. All authors contributed to the study design, critical revision of the manuscript and approved the final version.

## Supporting information

FIGURE S1 Summary of FE and RE models: Estimated SMD of HbA1c with 95% CI, between the low (exposed to glycaemic control) and high (unexposed) HbA1c groups during various time‐points of follow‐up (Sensitivity analysis—without Shalitin et al 2012)
FIGURE S2: HbA1c trajectories by studies and estimated overall trajectory (using population mean and SE)
FIGURE S3: HbA1c trajectories by studies (High v/s low)

**Additional File 1:** Search Strategy for research questions: Impact of early glycaemic control on long‐term HbA1c and risk of complications.
**Additional File 2:**: Electronic database search strategy
**Additional File 3:**: Inclusion and exclusion criteria for review of evidence on effect of early HbA1c levels on glycaemic trends and later complications
**Additional File 4:**: Details of data extracted from included studies
**Additional File 5:**: Quality assessment criteria

## References

[1] Hofer SE , Raile K , Frohlich‐Reiterer E , et al. Tracking of metabolic control from childhood to young adulthood in type 1 diabetes. J Pediatr. 2014;165(5):956‐61.e1‐2. 25151197 10.1016/j.jpeds.2014.07.001

[2] Edge JA , James T , Shine B . Persistent individual tracking within overall improvement in HbA1c in a UK paediatric diabetes clinic over 15 years. Diabet Med. 2010;27(11):1284‐1288. 20950387 10.1111/j.1464-5491.2010.03057.x

[3] Chiang JL , Maahs DM , Garvey KC , et al. Type 1 diabetes in children and adolescents: a position statement by the American Diabetes Association. Diabetes Care. 2018;41(9):2026‐2044. 30093549 10.2337/dci18-0023 PMC6105320

[4] Speight J , Amiel SA , Bradley C , et al. Long‐term biomedical and psychosocial outcomes following DAFNE (dose adjustment for Normal eating) structured education to promote intensive insulin therapy in adults with sub‐optimally controlled type 1 diabetes. Diabetes Res Clin Pract. 2010;89(1):22‐29. 20399523 10.1016/j.diabres.2010.03.017

[5] Brorsson AL , Viklund G , Ortqvist E , Lindholm Olinder A . Does treatment with an insulin pump improve glycaemic control in children and adolescents with type 1 diabetes? A retrospective case‐control study. Pediatr Diabetes. 2015;16(7):546‐553. 25327782 10.1111/pedi.12209

[6] The effect of intensive treatment of diabetes on the development and progression of long‐term complications in insulin‐dependent diabetes mellitus . The diabetes control and complications trial research group. N Engl J Med. 1993;329(14):977‐986. 8366922 10.1056/NEJM199309303291401

[7] Effect of intensive diabetes treatment on the development and progression of long‐term complications in adolescents with insulin‐dependent diabetes mellitus: diabetes control and complications trial. J Pediatr. 1994;125(2):177‐188. 8040759 10.1016/s0022-3476(94)70190-3

[8] Harjutsalo V , Maric‐Bilkan C , Forsblom C , Groop PH . Impact of sex and age at onset of diabetes on mortality from ischemic heart disease in patients with type 1 diabetes. Diabetes Care. 2014;37(1):144‐148. 24062319 10.2337/dc13-0377

[9] Amiel SA , Sherwin RS , Simonson DC , Lauritano AA , Tamborlane WV . Impaired insulin action in puberty. A contributing factor to poor glycemic control in adolescents with diabetes. N Engl J Med. 1986;315(4):215‐219. 3523245 10.1056/NEJM198607243150402

[10] Helgeson VS , Siminerio L , Escobar O , Becker D . Predictors of metabolic control among adolescents with diabetes: a 4‐year longitudinal study. J Pediatr Psychol. 2009;34(3):254‐270. 18667479 10.1093/jpepsy/jsn079 PMC2657034

[11] Wisting L , Froisland DH , Skrivarhaug T , Dahl‐Jorgensen K , Ro O . Disturbed eating behavior and omission of insulin in adolescents receiving intensified insulin treatment: a nationwide population‐based study. Diabetes Care. 2013;36(11):3382‐3387. 23963896 10.2337/dc13-0431 PMC3816868

[12] Meigs JB , Nathan DM , Cupples LA , Wilson PWF , Singer DE . Tracking of glycated hemoglobin in the original cohort of the Framingham heart study. J Clin Epidemiol. 1996;49(4):411‐417. 8621991 10.1016/0895-4356(95)00513-7

[13] Hamman RF , Bell RA , Dabelea D , et al. The SEARCH for diabetes in youth study: rationale, findings, and future directions. Diabetes Care. 2014;37(12):3336‐3344. 25414389 10.2337/dc14-0574 PMC4237981

[14] Rosenbauer J , Dost A , Karges B , et al. Improved metabolic control in children and adolescents with type 1 diabetes: a trend analysis using prospective multicenter data from Germany and Austria. Diabetes Care. 2012;35(1):80‐86. 22074726 10.2337/dc11-0993 PMC3241332

[15] Dovc K , Telic SS , Lusa L , et al. Improved metabolic control in pediatric patients with type 1 diabetes: a nationwide prospective 12‐year time trends analysis. Diabetes Technol Ther. 2014;16(1):33‐40. 24131373 10.1089/dia.2013.0182 PMC3887404

[16] Nathan DM , Turgeon H , Regan S . Relationship between glycated haemoglobin levels and mean glucose levels over time. Diabetologia. 2007;50(11):2239‐2244. 17851648 10.1007/s00125-007-0803-0 PMC8752566

[17] Svensson M , Eriksson JW , Dahlquist G . Early glycemic control, age at onset, and development of microvascular complications in childhood‐onset type 1 diabetes: a population‐based study in northern Sweden. Diabetes Care. 2004;27(4):955‐962. 15047655 10.2337/diacare.27.4.955

[18] Nirantharakumar K , Mohammed N , Toulis KA , Thomas GN , Narendran P . Clinically meaningful and lasting HbA1c improvement rarely occurs after 5 years of type 1 diabetes: an argument for early, targeted and aggressive intervention following diagnosis. Diabetologia. 2018;61(5):1064‐1070. 29478098 10.1007/s00125-018-4574-6 PMC6448997

[19] Mazarello Paes V , Charalampopoulos D , Khanolkar AR , et al. Protocol for systematic review of evidence on the determinants and influence of early glycaemic control in childhood‐onset type 1 diabetes. Syst Rev. 2015;4(1):159. 26563100 10.1186/s13643-015-0146-8 PMC4643492

[20] EPPI . Evidence for Policy and Practice Information (EPPI) and Co‐ordinating Centre, IoE, University of London EPPI ‐Centre Methods for Conducting Systematic Reviews http://eppi.ioe.ac.uk/cms/Default.aspx?tabid=184 last accessed September 15, 2018. 2018.

[21] Liberati A , Altman DG , Tetzlaff J , et al. The PRISMA statement for reporting systematic reviews and meta‐analyses of studies that evaluate health care interventions: explanation and elaboration. PLoS Med. 2009;6(7):e1000100. 19621070 10.1371/journal.pmed.1000100 PMC2707010

[22] Watson PF , Petrie A . Method agreement analysis: a review of correct methodology. Theriogenology. 2010;73(9):1167‐1179. 20138353 10.1016/j.theriogenology.2010.01.003

[23] Higgins JP, Green S, eds. Cochrane Handbook for Systematic Review of Interventions. Chichester: Wiley‐Blackwell; 2008.

[24] Samuelsson U , Steineck I , Gubbjornsdottir S . A high mean‐HbA1c value 3‐15 months after diagnosis of type 1 diabetes in childhood is related to metabolic control, macroalbuminuria, and retinopathy in early adulthood‐‐a pilot study using two nation‐wide population based quality registries. Pediatr Diabetes. 2014;15(3):229‐235. 24119008 10.1111/pedi.12085

[25] Sutton AJ , Higgins JPT . Recent developments in meta‐analysis. Stat Med. 2008;27(5):625‐650. 17590884 10.1002/sim.2934

[26] Higgins JPT , Thompson SG . Quantifying heterogeneity in a meta‐analysis. Stat Med. 2002;21(11):1539‐1558. 12111919 10.1002/sim.1186

[27] Lawes T , Franklin V , Farmer G . HbA1c tracking and bio‐psychosocial determinants of glycaemic control in children and adolescents with type 1 diabetes: retrospective cohort study and multilevel analysis. Pediatr Diabetes. 2014;15(5):372‐383. 24279611 10.1111/pedi.12100

[28] Shalitin S , Phillip M . Which factors predict glycemic control in children diagnosed with type 1 diabetes before 6.5 years of age? Acta Diabetol. 2012;49(5):355‐362. 21866397 10.1007/s00592-011-0321-x

[29] Clements MA , Lind M , Raman S , et al. Age at diagnosis predicts deterioration in glycaemic control among children and adolescents with type 1 diabetes. BMJ Open Diabetes Res Care. 2014;2(1):e000039. 10.1136/bmjdrc-2014-000039 PMC4212563 25452876

[30] Cabrera SM , Srivastava NT , Behzadi JM , Pottorff TM , Dimeglio LA , Walvoord EC . Long‐term glycemic control as a result of initial education for children with new onset type 1 diabetes: does the setting matter? Diabetes Educ. 2013;39(2):187‐194. 23427241 10.1177/0145721713475845 PMC4780749

[31] de Beaufort CE , Swift PGF , Skinner CT , et al. Continuing stability of center differences in pediatric diabetes care: do advances in diabetes treatment improve outcome? The Hvidoere study group on childhood diabetes. Diabetes Care. 2007;30(9):2245‐2250. 17540955 10.2337/dc07-0475

[32] Svensson J , Johannesen J , Mortensen HB , Nordly S , On behalf of The Danish Childhood Diabetes Registry . Improved metabolic outcome in a Danish diabetic paediatric population aged 0‐18 yr: results from a nationwide continuous registration. Pediatr Diabetes. 2009;10(7):461‐467. 19175901 10.1111/j.1399-5448.2008.00460.x

[33] Dabadghao P , Vidmar S , Cameron FJ . Deteriorating diabetic control through adolescence‐do the origins lie in childhood? Diabet Med. 2001;18(11):889‐894. 11703433 10.1046/j.1464-5491.2001.00593.x

[34] Yudkin JS , Forrest RD , Jackson CA , Ryle AJ , Davie S , Gould BJ . Unexplained variability of glycated haemoglobin in non‐diabetic subjects not related to glycaemia. Diabetologia. 1990;33(4):208‐215. 2347434 10.1007/BF00404798

[35] Kilpatrick ES , Rigby AS , Atkin SL . Variability in the relationship between mean plasma glucose and HbA1c: implications for the assessment of glycemic control. Clin Chem. 2007;53(5):897‐901. 17384010 10.1373/clinchem.2006.079756

[36] Hempe JM , Gomez R , McCarter RJ Jr , Chalew SA . High and low hemoglobin glycation phenotypes in type 1 diabetes: a challenge for interpretation of glycemic control. J Diabetes Complications. 2002;16(5):313‐320. 12200073 10.1016/s1056-8727(01)00227-6

[37] Luyckx K , Seiffge‐Krenke I . Continuity and change in glycemic control trajectories from adolescence to emerging adulthood: relationships with family climate and self‐concept in type 1 diabetes. Diabetes Care. 2009;32(5):797‐801. 19228859 10.2337/dc08-1990 PMC2671120

[38] Bryden KS , Dunger DB , Mayou RA , Peveler RC , Neil HAW . Poor prognosis of young adults with type 1 diabetes: a longitudinal study. Diabetes Care. 2003;26(4):1052‐1057. 12663572 10.2337/diacare.26.4.1052

[39] Nathan DM , Bayless M , Cleary P , et al. Diabetes control and complications trial/epidemiology of diabetes interventions and complications study at 30years: advances and contributions. Diabetes. 2013;62(12):3976‐3986. 24264395 10.2337/db13-1093 PMC3837056

[40] Ali MK , Bullard KM , Saaddine JB , Cowie CC , Imperatore G , Gregg EW . Achievement of goals in U.S. diabetes care, 1999‐2010. N Engl J Med. 2013;368(17):1613‐1624. 23614587 10.1056/NEJMsa1213829

[41] Beck RW , Tamborlane WV , Bergenstal RM , Miller KM , DuBose SN , Hall CA . The T1D exchange clinic registry. J Clin Endocrinol Metab. 2012;97(12):4383‐4389. 22996145 10.1210/jc.2012-1561

[42] RCPCH. National Paediatric Diabetes Audit Report 2014‐15. Care processes and outcomes 2016. Available from: http://www.rcpch.ac.uk/sites/default/files/page/NPDA%20Report%202014-15%20v5.2%20sent%20to%20HQIP%2025.05.2016.pdf.

[43] Olsen BS , Sjolie AK , Hougaard P , et al. The significance of the prepubertal diabetes duration for the development of retinopathy and nephropathy in patients with type 1 diabetes. J Diabetes Complications. 2004;18(3):160‐164. 15145327 10.1016/S1056-8727(03)00073-4

[44] Fredheim S , Johannesen J , Johansen A , et al. Diabetic ketoacidosis at the onset of type 1 diabetes is associated with future HbA1c levels. Diabetologia. 2013;56(5):995‐1003. 23389397 10.1007/s00125-013-2850-z

[45] Larsen J , Brekke M , Sandvik L , Arnesen H , Hanssen KF , Dahl‐Jorgensen K . Silent coronary atheromatosis in type 1 diabetic patients and its relation to long‐term glycemic control. Diabetes. 2002;51(8):2637‐2641. 12145181 10.2337/diabetes.51.8.2637

[46] Dabelea D , Kinney G , Snell‐Bergeon JK , et al. Effect of type 1 diabetes on the gender difference in coronary artery calcification: a role for insulin resistance? The coronary artery calcification in type 1 diabetes (CACTI) study. Diabetes. 2003;52(11):2833‐2839. 14578303 10.2337/diabetes.52.11.2833

[47] Herrett E , Shah AD , Boggon R , et al. Completeness and diagnostic validity of recording acute myocardial infarction events in primary care, hospital care, disease registry, and national mortality records: cohort study. BMJ. Clin Res Ed. 2013;346:f2350. 10.1136/bmj.f2350 PMC3898411 23692896

[48] Pajunen P , Taskinen MR , Nieminen MS , Syvanne M . Angiographic severity and extent of coronary artery disease in patients with type 1 diabetes mellitus. Am J Cardiol. 2000;86(10):1080‐1085. 11074203 10.1016/s0002-9149(00)01163-2

[49] Nathan DM , Lachin J , Cleary P , et al. Intensive diabetes therapy and carotid intima‐media thickness in type 1 diabetes mellitus. N Engl J Med. 2003;348(23):2294‐2303. 12788993 10.1056/NEJMoa022314 PMC2701300

[50] Nathan DM , Cleary PA , Backlund JY , et al. Intensive diabetes treatment and cardiovascular disease in patients with type 1 diabetes. N Engl J Med. 2005;353(25):2643‐2653. 16371630 10.1056/NEJMoa052187 PMC2637991

[51] Dahl‐Jorgensen K , Larsen JR , Hanssen KF . Atherosclerosis in childhood and adolescent type 1 diabetes: early disease, early treatment? Diabetologia. 2005;48(8):1445‐1453. 15971059 10.1007/s00125-005-1832-1

[52] Giannattasio C , Failla M , Piperno A , et al. Early impairment of large artery structure and function in type I diabetes mellitus. Diabetologia. 1999;42(8):987‐994. 10491759 10.1007/s001250051257

[53] Schwab KO , Doerfer J , Hecker W , et al. Spectrum and prevalence of atherogenic risk factors in 27,358 children, adolescents, and young adults with type 1 diabetes: cross‐sectional data from the German diabetes documentation and quality management system (DPV). Diabetes Care. 2006;29(2):218‐225. 16443863 10.2337/diacare.29.02.06.dc05-0724

[54] Amin R , Frystyk J , Ong K , Dalton RN , Flyvbjerg A , Dunger DB . The development of microalbuminuria is associated with raised longitudinal adiponectin levels in female but not male adolescent patients with type 1 diabetes. Diabetologia. 2008;51(9):1707‐1713. 18622594 10.1007/s00125-008-1081-1

[55] Adolescent type 1 Diabetes Cardio‐renal Intervention Trial (AdDIT).BMC Pediatr . 2009 Dec 17;9:79. 10.1186/1471-2431-9-79. PMC2814806 20017932

