# Supplementary material for: Effect of early glycemic control on HbA1c tracking and development of vascular complications after 5 years of childhood onset type 1 diabetes: Systematic review and meta‐analysis
Source: Pediatr Diabetes. 2019 Apr 24;20(5):494–509. doi: 10.1111/pedi.12850 (PMC6701989; doi:10.1111/pedi.12850)
Supplement: Supplementary file 1 — FIGURE S1 Summary of FE and RE models: Estimated SMD of HbA1c with 95% CI, between the low (exposed to glycaemic control) and high (unexposed) HbA1c groups during various time‐points of follow‐up (Sensitivity analysis—without Shalitin et al 2012) FIGURE S2: HbA1c trajectories by studies and estimated overall trajectory (using population mean and SE) FIGURE S3: HbA1c trajectories by studies (High v/s low) [file PEDI-20-494-s001.docx]

**Supplementary Fig 1:** **Summary of FE & RE models: Estimated SMD of HbA1c with 95%CI, between the low (exposed to glycaemic control) and high (unexposed) HbA1c groups during various time-points of follow-up (Sensitivity analysis - without Shalitin et al 2012)**

**SMD: standardised mean difference; CI: confidence interval; N: number of participants; SD: standard deviation; I-V: inverse variance; D+L: DerSimonian and Laird**

**Supplementary Fig 2: HbA1c trajectories by studies and estimated overall trajectory (using population mean and standard error)**

**Supplementary Fig 3: HbA1c trajectories by studies (High v/s low)**
