# Supplementary material for: Effect of early glycemic control on HbA1c tracking and development of vascular complications after 5 years of childhood onset type 1 diabetes: Systematic review and meta‐analysis
Source: Pediatr Diabetes. 2019 Apr 24;20(5):494–509. doi: 10.1111/pedi.12850 (PMC6701989; doi:10.1111/pedi.12850)
Supplement: Supplementary file 2 — Additional File 1: Search Strategy for research questions: Impact of early glycaemic control on long‐term HbA1c and risk of complications. Additional File 2:: Electronic database search strategy Additional File 3:: Inclusion and exclusion criteria for review of evidence on effect of early HbA1c levels on glycaemic trends and later complications Additional File 4:: Details of data extracted from included studies Additional File 5:: Quality assessment criteria [file PEDI-20-494-s002.docx]

**Additional File 1: Search Strategy for research questions: Impact of early glycaemic control on long-term HbA1c and risk of complications.**

| **Population** | **Exposure** | **Outcome** |
| --- | --- | --- |
| Childhood or paediatric onset diabetes or juvenile diabetes diagnosis or newly diagnosed children or young persons or young people or children or young or adolescent or teen or youth or adult T1D patient or type 1 diabetes or T1D or type 1 diabetes mellitus or T1DM or DM1 or type 1 or IDDM or insulin dependent or non-insulin dependent or childhood onset diabetes or childhood onset T1D or auto-immune or autoimmune or sudden onset or uncontrolled or labile or brittle | Early diabetes control or HbA1c trajectories or HbA1c trends or glycaemic trajectories or glycosylated or HbA1c or A1c or Hemoglobin A or HbA(1c) level or glycaemic control or glucose control or diabetes control or early intensive intervention or intensive or conventional or standard or regular or optimised or tight control or strict control or usual or routine or therapy or treatment or intervention or management or insulin use or injection or dose insulin injections or intensive therapy or insulin pump | Diabetic or diabetes complications or complications or side effects or adverse events or acute complications or chronic complications or  glycaemia or hyper glycaemia or hypo glycaemia or ketosis or diabetic ketoacidosis or DKA or nonketotic hyperosmolar coma or insulin resistance or autoimmune disease or urine albumin or urine albumin creatinine ratio or urine albumin excretion or microalbuminuria or macroalbuminuria or renal disease or diabetic nephropathy or nephropathy or dialysis or foot ulcer or amputation or retinopathy or blindness or vascular disease or vascular complications or microvascular disease or microvascular complications or macrovascular disease or macrovascular complications or cardiovascular disease or MI or myocardial infarction or stroke or coronary artery disease or cerebrovascular disease or peripheral vascular disease or blood pressure or BP or statin or death or mortality or Pathology or metabolism or metabolic memory |

**Additional File 2: Electronic database search strategy**

|  | **Scopus (via Elsevier) (Original: 17/12/2014; Updated: 17/02/2017)** |
| --- | --- |
| 1. | ( TITLE-ABS-KEY ( ( {early intensive} OR tight OR glycemic OR glycaemic OR glucose OR diabetes OR strict ) W/2 control ) OR TITLE-ABS-KEY ( insulin W/2 ( use* OR injection* OR dose* OR pump* ) ) OR TITLE-ABS-KEY ( glycosylat* OR {HbA1c} OR a1c OR hemoglobin a OR haemoglobin OR {HbA(1c)} ) OR TITLE-ABS-KEY ( ( intensive OR conventional OR standard OR regular OR optimised OR usual OR routine ) W/2 ( care OR treatment OR therapy OR intervention OR management ) ) OR TITLE-ABS-KEY ( hyperglycaemia OR hypoglycaemia ) ) AND ( TITLE-ABS-KEY OR ( {Diabetes complication*} OR {side effects} OR {adverse events} OR glycemia OR glycaemia OR {hyper glycemia} OR {hyper glycaemia} OR hyperglycemia OR hyperglycaemia OR {hypo glycemia} OR {hypo glycaemia} OR hypoglycemia OR hypoglycaemia ) OR TITLE-ABS-KEY ( ketosis OR {diabetic ketoacidosis} OR dka OR {nonketotic hyperosmolar coma} OR {insulin resistance} OR {autoimmune disease*} OR {auto immune disease} ) OR TITLE-ABS-KEY ( {urine albumin} OR microalbuminaria OR macroalbuminuria OR {renal disease*} OR {kidney disease*} OR {diabetic nephropathy} OR nephropathy OR dialysis ) OR TITLE-ABS-KEY ( {foot ulcer} OR amputation ) OR TITLE-ABS-KEY ( retinopathy OR blindness OR {cardiovascular disease*} OR mi OR {myocardial infarction*} OR stroke* OR {coronary artery disease*} ) OR TITLE-ABS-KEY ( {cerebrovascular disease*} OR {peripheral vascular disease*} OR {blood pressure} OR bp OR statin* OR death OR mortality ) ) AND ( TITLE-ABS-KEY ( pediatric OR paediatric OR child* OR {young people} OR youth OR {young adult*} OR juvenile OR {insulin dependent} OR labile OR brittle OR {sudden onset} OR autoimmune OR {auto immune} OR {non insulin dependent} OR uncontrolled OR {newly diagnosed} OR {new diagnosis} OR {inception diabetes} ) ) AND ( TITLE-ABS-KEY ( dm1 OR {diabetes mellitus 1} OR {diabetes mellitus} W/2 {type 1} ) OR t1d OR t1dm OR iddmor {type 1} ) AND TITLE-ABS-KEY ( metabolism OR {metabolic memory} ) |
| 2 | TITLE-ABS-KEY ( metabolism OR {metabolic memory} ) |
| 3 | (TITLE-ABS-KEY or ({Diabetes complication*} or {side effects} or {adverse events} or glycemia or glycaemia or {hyper glycemia} or {hyper glycaemia} or hyperglycemia or hyperglycaemia or {hypo glycemia} or {hypo glycaemia} or hypoglycemia or hypoglycaemia) or TITLE-ABS-KEY (ketosis or {diabetic ketoacidosis} or DKA or {nonketotic hyperosmolar coma} or {insulin resistance} or {autoimmune disease*} or {auto immune disease}) or TITLE-ABS-KEY ({urine albumin} or microalbuminaria or macroalbuminuria or {renal disease*} or {kidney disease*} or {diabetic nephropathy} or nephropathy or dialysis) or TITLE-ABS-KEY ({foot ulcer} or amputation) or TITLE-ABS-KEY (retinopathy or blindness or {cardiovascular disease*} or MI or {myocardial infarction*} or stroke* or {coronary artery disease*}) or TITLE-ABS-KEY ({cerebrovascular disease*} or {peripheral vascular disease*} or {blood pressure} or BP or statin* or death or mortality)) |
| 4 | (TITLE-ABS-KEY (({early intensive} or tight or glycemic or glycaemic or glucose or diabetes or strict) W/2 control) OR TITLE-ABS-KEY (insulin W/2 (use* or injection* or dose* or pump*)) OR TITLE-ABS-KEY (glycosylat* or {HbA1c} or A1c or Hemoglobin A or haemoglobin or {HbA(1c)}) OR TITLE-ABS-KEY((intensive or conventional or standard or regular or optimised or usual or routine) W/2 (care or treatment or therapy or intervention or management)) OR TITLE-ABS-KEY (hyperglycaemia or hypoglycaemia)) |
| 5 | ( TITLE-ABS-KEY ( pediatric OR paediatric OR child* OR {young people} OR youth OR {young adult*} OR juvenile OR {insulin dependent} OR labile OR brittle OR {sudden onset} OR autoimmune OR {auto immune} OR {non insulin dependent} OR uncontrolled OR {newly diagnosed} OR {new diagnosis} OR {inception diabetes} ) ) |
| 6 | ( TITLE-ABS-KEY ( dm1 OR {diabetes mellitus 1} OR {diabetes mellitus} W/2 {type 1} ) OR t1d OR t1dm OR iddmor {type 1} ) |

|  | **Cochrane Library (Original: 17/12/2014; Updated: 17/02/2017)** |
| --- | --- |
| #1 | MeSH descriptor: [Diabetes Mellitus, Type 1] explode all trees |
| #2 | DM1 or diabetes mellitus 1 or diabetes mellitus type 1 or T1D or T1DM or IDDM |
| #3 | type 1 or paediatric or child or young people or youth or young adults or juvenile or insulin dependent or labile or brittle or sudden onset or autoimmune or auto immune or non insulin dependent or uncontrolled or newly diagnosed or new diagnosis or inception diabetes |
| #4 | #1 or #2 or #3 |
| #5 | MeSH descriptor: [Blood Glucose] explode all trees |
| #6 | MeSH descriptor: [Hemoglobin A, Glycosylated] explode all trees |
| #7 | MeSH descriptor: [Hypoglycemia] explode all trees |
| #8 | MeSH descriptor: [Hyperglycemia] explode all trees |
| #9 | #5 or #6 or #7 or #8 |
| #10 | early intensive or tight or glycemic or glucose or diabetes or strict control |
| #11 | insulin use or injection or dose or pump |
| #12 | glycosylate or HbA1c or A1c or Hemoglobin A or HbA1c |
| #13 | intensive or conventional or standard or regular or optimised or usual or routine care or treatment or therapy or intervention or management |
| #14 | #9 or #10 or #11 or #12 or #13 |
| #15 | MeSH descriptor: [Diabetes Complications] explode all trees |
| #16 | adverse effects or complications |
| #17 | MeSH descriptor: [Ketosis] explode all trees |
| #18 | MeSH descriptor: [Insulin Resistance] explode all trees |
| #19 | MeSH descriptor: [Autoimmune Diseases] explode all trees |
| #20 | MeSH descriptor: [Albuminuria] explode all trees |
| #21 | MeSH descriptor: [Kidney Diseases] explode all trees |
| #22 | MeSH descriptor: [Dialysis] explode all trees |
| #23 | MeSH descriptor: [Blindness] explode all trees |
| #24 | MeSH descriptor: [Cardiovascular Diseases] explode all trees |
| #25 | MeSH descriptor: [Cerebrovascular Disorders] explode all trees |
| #26 | MeSH descriptor: [Blood Pressure] explode all trees |
| #27 | MeSH descriptor: [Hydroxymethylglutaryl-CoA Reductase Inhibitors] explode all trees |
| #28 | MeSH descriptor: [Mortality] explode all trees |
| #29 | Diabetes complications or side effects or adverse events or glycaemia or hyper glycaemia or hypo glycaemia or ketosis or diabetic ketoacidosis or DKA or nonketotic hyperosmolar coma or insulin resistance or autoimmune disease or urine albumin or urine albumin creatinine ratio or urine albumin excretion or microalbuminuria or macroalbuminuria or renal disease or diabetic nephropathy or nephropathy or dialysis or foot ulcer or amputation or retinopathy or blindness or cardiovascular disease or MI or myocardial infarction or stroke or coronary artery disease or cerebrovascular disease or peripheral vascular disease or blood pressure or BP or statin or death or mortality |
| #30 | #15 or #16 or #17 or #18 or #19 or #20 or #21 or #22 or #23 or #24 or #25 or #26 or #27 or #28 or #29 |
| #31 | metabolism |
| #32 | metabolic memory |
| #33 | #31 or #32 |
| #34 | #4 and #9 and #14 and #30 and #33 |

|  | **CINAHL Plus (via EBSCO) (Original: 16/12/2014; Updated: 17/02/2017)** |
| --- | --- |
| S34 | S4 AND S14 AND S30 AND S33 |
| S33 | S31 OR S32 |
| S32 | "metabolic memory" |
| S31 | MJ metabolism |
| S30 | S15 OR S16 OR S17 OR S18 OR S19 OR S20 OR S21 OR S22 OR S23 OR S24 OR S25 OR S26 OR S27 OR S28 OR S29 |
| S29 | diabetes complication or diabetes complication* or side effects or adverse events or glyc#emia or hyper glyc#emia or hyperglyc#emia or hypo glyc#emia or hypoglyc#emia or ketosis or diabetic ketoacidosis or DKA or nonketotic hyperosmolar coma or insulin resistance or autoimmune disease* or urine albumin or microalbuminaria or macroalbuminuria or renal disease* or kidney disease* or diabetic nephropathy or nephropathy or dialysis or foot ulcer or amputation or retinopathy or blindness or cardiovascular disease* or MI or myocardial infarction* or stroke* or coronary artery disease* or cerebrovascular disease* or peripheral vascular disease* or blood pressure or BP or statin* or death or mortality |
| S28 | (MH "mortality+") |
| S27 | (MH "statins+") |
| S26 | (MH "blood pressure+") |
| S25 | (MH "cerebrovascular disorders+") |
| S24 | (MH "stroke+") |
| S23 | (MH "cardiovascular diseases+") |
| S22 | (MH "blindness+") |
| S21 | (MH "dialysis+") |
| S20 | (MH "kidney diseases+") |
| S19 | (MH "Albuminuria") |
| S18 | (MH autoimmune diseases+) |
| S17 | (MH insulin resistance+) |
| S16 | (MH "diabetic angiopathies+") OR (MH "diabetic cardiomyopathies") OR (MH "diabetic coma+") OR (MH "diabetic ketoacidosis") OR (MH "diabetic neuropathies+") |
| S15 | (MH "diabetes mellitus/co") |
| S14 | S5 OR S6 OR S7 OR S8 OR S9 OR S10 OR S11 OR S12 OR S13 |
| S13 | (intensive OR conventional OR standard OR regular OR optimi#ed OR usual OR routine) N2 (care OR treatment OR therapy OR intervention OR management) |
| S12 | glycosylat* OR HbA1c OR A1c OR H#emoglobin A OR HbA#1c |
| S11 | insulin N2 (use* OR injection* OR dose* OR pump*) |
| S10 | (“early intensive” OR tight OR glyc#emic OR glucose OR diabetes or strict) N2 control) |
| S9 | (MH "Hyperglycemia+") |
| S8 | (MH "Hypoglycemia+") |
| S7 | MH blood glucose |
| S6 | MH hemoglobin A, glycosylated |
| S5 | (MH "Glycemic Control") |
| S4 | S1 OR S2 OR S3 |
| S3 | ("type 1" OR p#ediatric OR child* OR "young people" OR youth OR "young adult" OR juvenile OR "insulin dependent" OR labile OR brittle OR "sudden onset" OR autoimmune OR "auto immune" OR "non insulin dependent" OR uncontrolled OR "newly diagnosed" OR "new diagnosis" OR inception) N5 diabetes |
| S2 | DM1 OR "diabetes mellitus 1" OR ("diabetes mellitus" N2 type 1) OR T1D or T1DM or IDDM |
| S1 | (MH "Diabetes Mellitus, Type 1+") |

|  | **Web of Science (via Thomson Reuters) (Original: 16/12/2014; Updated: 17/02/2017)** |
| --- | --- |
| 1 | TOPIC: ((DM1 OR "diabetes mellitus 1" OR ("diabetes mellitus" NEAR/2 "type 1") OR T1D or T1DM or IDDM) OR TOPIC: (("type 1" OR p$ediatric OR child* OR "young people" OR youth OR "young adult" OR juvenile OR "insulin dependent" OR labile OR brittle OR "sudden onset" OR autoimmune OR "auto immune" OR "non insulin dependent" OR uncontrolled OR "newly diagnosed" OR "new diagnosis" OR inception) NEAR/5 diabetes))  Indexes=SCI-EXPANDED, SSCI, A&HCI, CPCI-S, CPCI-SSH, BKCI-S, BKCI-SSH, CCR-EXPANDED, IC Timespan=All years |
| 2 | TOPIC: (TOPIC: ((“early intensive” OR tight OR glyc$emic OR glucose OR diabetes or strict) NEAR/2 control) OR TOPIC: (insulin NEAR/2 (use* OR injection* OR dose* OR pump*)) OR TOPIC: (glycosylat* OR HbA1c OR A1c OR H$emoglobin A OR HbA$1c) OR TOPIC: ((intensive OR conventional OR standard OR regular OR optimi$ed OR usual OR routine) NEAR/2 (care OR treatment OR therapy OR intervention OR management)) OR TOPIC: (hyperglyc$emia OR hypoglyc$emia))  Indexes=SCI-EXPANDED, SSCI, A&HCI, CPCI-S, CPCI-SSH, BKCI-S, BKCI-SSH, CCR-EXPANDED, IC Timespan=All years |
| 3 | TOPIC: (TOPIC: (“Diabetes complication*” OR “side effects” OR “adverse events” OR glyc$emia OR “hyper glyc$emia” OR hyperglyc$emia OR “hypo glyc$emia” OR hypoglyc$emia OR ketosis OR “diabetic ketoacidosis” OR DKA OR “nonketotic hyperosmolar coma” OR “insulin resistance” OR “autoimmune disease*” OR “auto immune disease” OR “urine albumin” OR microalbuminaria OR macroalbuminuria OR “renal disease*” OR “kidney disease*” OR nephropathy OR dialysis OR “foot ulcer” OR amputation OR retinopathy OR blindness OR “cardiovascular disease*” OR MI OR “myocardial infarction*” OR stroke* OR “coronary artery disease*” OR “cerebrovascular disease*” OR “peripheral vascular disease*” OR “blood pressure” OR BP OR statin* OR death OR mortality))  Indexes=SCI-EXPANDED, SSCI, A&HCI, CPCI-S, CPCI-SSH, BKCI-S, BKCI-SSH, CCR-EXPANDED, IC Timespan=All years |
| 4 | TOPIC: (TOPIC: (metabolism OR "metabolic memory" OR metabolic))  Indexes=SCI-EXPANDED, SSCI, A&HCI, CPCI-S, CPCI-SSH, BKCI-S, BKCI-SSH, CCR-EXPANDED, IC Timespan=All years |
| 5 | #4 AND #3 AND #2 AND #1  DocType=All document types; Language=All languages; |

|  | **EMBASE (via OVID) (Original: 16/12/2014; Updated: 17/02/2017)** |
| --- | --- |
| 1 | exp insulin dependent diabetes mellitus/ |
| 2 | (DM1 or diabetes mellitus 1 or (diabetes mellitus adj2 type 1) or T1D or T1DM or IDDM).mp. [mp=title, abstract, heading word, drug trade name, original title, device manufacturer, drug manufacturer, device trade name, keyword, floating subheading] |
| 3 | ((type 1 or p?ediatric or child* or young people or youth or young adults or juvenile or insulin dependent or labile or brittle or sudden onset or autoimmune or auto immune or non insulin dependent or uncontrolled or newly diagnosed or new diagnosis or inception) adj5 diabetes).mp. [mp=title, abstract, heading word, drug trade name, original title, device manufacturer, drug manufacturer, device trade name, keyword, floating subheading] |
| 4 | 1 or 2 or 3 |
| 5 | exp glycosylated hemoglobin/ |
| 6 | exp glucose blood level/ |
| 7 | exp hypoglycemia/ |
| 8 | exp hyperglycemia/ |
| 9 | ((early intensive or tight or glyc?emic or glucose or diabetes or strict) adj2 control).mp. [mp=title, abstract, heading word, drug trade name, original title, device manufacturer, drug manufacturer, device trade name, keyword, floating subheading] |
| 10 | (insulin adj2 (use* or injection* or dose* or pump*)).mp. [mp=title, abstract, heading word, drug trade name, original title, device manufacturer, drug manufacturer, device trade name, keyword, floating subheading] |
| 11 | (glycosylat* or HbA1c or A1c or H?emoglobin A or HbA?1c).mp. [mp=title, abstract, heading word, drug trade name, original title, device manufacturer, drug manufacturer, device trade name, keyword, floating subheading] |
| 12 | ((intensive or conventional or standard or regular or optimi?ed or usual or routine) adj2 (care or treatment or therapy or intervention or management)).mp. [mp=title, abstract, heading word, drug trade name, original title, device manufacturer, drug manufacturer, device trade name, keyword, floating subheading] |
| 13 | 5 or 6 or 7 or 8 or 9 or 10 or 11 or 12 |
| 14 | diabetic angiopathy/ or diabetic cardiomyopathy/ or diabetic coma/ or diabetic foot/ or diabetic hypertension/ or diabetic ketoacidosis/ or diabetic macular edema/ or diabetic nephropathy/ or diabetic neuropathy/ or diabetic obesity/ or diabetic retinopathy/ or impaired glucose tolerance/ or "maternally inherited diabetes and deafness"/ or nonketotic diabetic coma/ or wolfram syndrome/ |
| 15 | exp diabetes mellitus/co [Complication] |
| 16 | exp diabetes mellitus/si [Side Effect] |
| 17 | exp insulin resistance/ |
| 18 | exp autoimmune disease/ |
| 19 | exp albuminuria/ |
| 20 | exp kidney disease/ |
| 21 | exp dialysis/ |
| 22 | exp blindness/ |
| 23 | exp cardiovascular disease/ |
| 24 | exp cerebrovascular disease/ |
| 25 | exp blood pressure/ |
| 26 | exp hydroxymethylglutaryl coenzyme A reductase inhibitor/ |
| 27 | exp mortality/ |
| 28 | (Diabetes complication* or side effects or adverse events or glyc?emia or hyper glyc?emia or hyperglyc?emia or hypo glyc?emia or hypoglyc?emia or ketosis or diabetic ketoacidosis or DKA or nonketotic hyperosmolar coma or insulin resistance or autoimmune disease* or urine albumin or microalbuminaria or macroalbuminuria or renal disease* or kidney disease* or diabetic nephropathy or nephropathy or dialysis or foot ulcer or amputation or retinopathy or blindness or cardiovascular disease* or MI or myocardial infarction* or stroke* or coronary artery disease* or cerebrovascular disease* or peripheral vascular disease* or blood pressure or BP or statin* or death or mortality).mp. [mp=title, abstract, heading word, drug trade name, original title, device manufacturer, drug manufacturer, device trade name, keyword, floating subheading] |
| 29 | 14 or 15 or 16 or 17 or 18 or 19 or 20 or 21 or 22 or 23 or 24 or 25 or 26 or 27 or 28 |
| 30 | memory/ |
| 31 | metabolic memory.mp. [mp=title, abstract, heading word, drug trade name, original title, device manufacturer, drug manufacturer, device trade name, keyword, floating subheading] |
| 32 | 30 or 31 |
| 33 | 4 and 13 and 29 and 32 |

|  | **Medline (via OVID) (Original: 16/12/2014; Updated: 17/02/2017)** |
| --- | --- |
| 1 | exp Diabetes Mellitus, Type 1/ |
| 2 | (((DM1 or diabetes mellitus 1 or diabetes mellitus) adj2 type 1) or T1D or T1DM or IDDM).mp. [mp=title, abstract, original title, name of substance word, subject heading word, keyword heading word, protocol supplementary concept word, rare disease supplementary concept word, unique identifier, synonyms] |
| 3 | ((type 1 or p?ediatric or child* or young people or youth or young adults or juvenile or insulin dependent or labile or brittle or sudden onset or autoimmune or auto immune or non insulin dependent or uncontrolled or newly diagnosed or new diagnosis or inception) adj5 diabetes).mp. [mp=title, abstract, original title, name of substance word, subject heading word, keyword heading word, protocol supplementary concept word, rare disease supplementary concept word, unique identifier, synonyms] |
| 4 | 1 or 2 or 3 |
| 5 | exp Blood Glucose/ |
| 6 | hemoglobins/ or hemoglobin a, glycosylated/ |
| 7 | exp Hypoglycemia/ |
| 8 | exp Hyperglycemia/ |
| 9 | ((early intensive or tight or glyc?emic or glucose or diabetes or strict) adj2 control).mp. [mp=title, abstract, original title, name of substance word, subject heading word, keyword heading word, protocol supplementary concept word, rare disease supplementary concept word, unique identifier, synonyms] |
| 10 | (insulin adj2 (use* or injection* or dose* or pump*)).mp. [mp=title, abstract, original title, name of substance word, subject heading word, keyword heading word, protocol supplementary concept word, rare disease supplementary concept word, unique identifier, synonyms] |
| 11 | (glycosylat* or HbA1c or A1c or H?emoglobin A or HbA?1c).mp. [mp=title, abstract, original title, name of substance word, subject heading word, keyword heading word, protocol supplementary concept word, rare disease supplementary concept word, unique identifier, synonyms] |
| 12 | ((intensive or conventional or standard or regular or optimi?ed or usual or routine) adj2 (care or treatment or therapy or intervention or management)).mp. [mp=title, abstract, original title, name of substance word, subject heading word, keyword heading word, protocol supplementary concept word, rare disease supplementary concept word, unique identifier, synonyms] |
| 13 | 5 or 6 or 7 or 8 or 9 or 10 or 11 or 12 |
| 14 | exp Diabetes Complications/ |
| 15 | adverse effects.fs. |
| 16 | complications.fs. |
| 17 | exp Ketosis/ |
| 18 | exp Insulin Resistance/ |
| 19 | exp Autoimmune Diseases/ |
| 20 | exp Albuminuria/ |
| 21 | exp Kidney Diseases/ |
| 22 | exp Dialysis/ |
| 23 | exp Blindness/ |
| 24 | exp Cardiovascular Diseases/ |
| 25 | exp Cerebrovascular Disorders/ |
| 26 | exp Blood Pressure/ |
| 27 | exp Hydroxymethylglutaryl-CoA Reductase Inhibitors/ |
| 28 | exp Mortality/ |
| 29 | (Diabetes complications or side effects or adverse events or glycaemia or hyper glycaemia or hypo glycaemia or ketosis or diabetic ketoacidosis or DKA or nonketotic hyperosmolar coma or insulin resistance or autoimmune disease or urine albumin or urine albumin creatinine ratio or urine albumin excretion or microalbuminuria or macroalbuminuria or renal disease or diabetic nephropathy or nephropathy or dialysis or foot ulcer or amputation or retinopathy or blindness or cardiovascular disease or MI or myocardial infarction or stroke or coronary artery disease or cerebrovascular disease or peripheral vascular disease or blood pressure or BP or statin or death or mortality).mp. [mp=title, abstract, original title, name of substance word, subject heading word, keyword heading word, protocol supplementary concept word, rare disease supplementary concept word, unique identifier, synonyms] |
| 30 | 14 or 15 or 16 or 17 or 18 or 19 or 20 or 21 or 22 or 23 or 24 or 25 or 26 or 27 or 28 |
| 31 | 4 and 13 and 30 |
| 32 | metabolism.fs. |
| 33 | metabolic memory.mp. |
| 34 | 32 or 33 |
| 35 | 31 and 34 |

**Additional File 3: Inclusion and exclusion criteria for review of evidence on effect of early HbA1c levels on glycaemic trends and later complications**

| **Inclusion Criteria** | **Exclusion Criteria** |
| --- | --- |
| - Interventional studies (RCT’s and non-RCT’s) targeting glycaemic control (within 2 years of diagnosis of T1D) and described an association with health outcomes  - Non-intervention/observational i.e. cohort and cross sectional (XS) studies that quantified the association between early glycaemic control (within 2 years of diagnosis of T1D) AND risk of future complications in children and young people aged 0 to 19 years at baseline  - longitudinal studies with a follow-up of ≥ 5 years from diagnosis | - Non-human studies  - Selection of population based on other diseases/co-morbidities  - Adults aged more than 19 years at baseline.  - Studies on T2D  - Quantitative studies not reporting clinical outcomes  - Quantitative studies that measured glycaemic control but did not describe an association with outcome variables |

**Additional File 4: Details of data extracted from included studies**

| **Observational/Non-intervention studies (cross-sectional and longitudinal)** |
| --- |
| - Study id  - Author  - Year  - Country  - Age Range  - Average age  - Sex (Male: Female ratio)  - Ethnicity  - Socioeconomic status  - Design (Cross sectional/longitudinal)  - Number of participants  - Sample/recruitment e.g general population representative sample or specialist groups,  - Exposure examined  - Measurement of Exposure  - Measurement conducted by Level of glycaemic control  - Setting (home, primary care, secondary care)  - Outcome (HbA1c levels, complications, HbA1c tracking/metabolic memory - separate row for each outcome investigated),  - Measurement of outcome (objective)  - Analysis  - Effect  - Author email  - Comments |

**Additional File 5: Quality assessment criteria**

| **For observational (prospective/retrospective cohort and cross sectional) studies** |
| --- |
| Total quality assessment score (**maximum of 6**) was derived for fulfilment of following criteria:  1) More than 50 participants analysed;  2) Studies representing general population  3) Prospective study design  4) Adjusted/multivariate analysis  5) Objective measure of outcome  6) Objective measure of exposure. |
